# Vascular regeneration and blood flow recovery in glaucoma

**DOI:** 10.3389/fcell.2026.1851815

**Published:** 2026-06-03

**Authors:** Susannah Waxman, Adriana Di Polo

**Affiliations:** 1 Universite de Montreal, Montreal, QC, Canada; 2 Centre de Recherche du Centre Hospitalier de l'Universite de Montreal, Montreal, QC, Canada

**Keywords:** blood flow, glaucoma, neurodegenaration, neurovascular unit, regeneration, vascular

## Abstract

The retina and optic nerve rely on a tightly regulated neurovascular unit that sustains the highly dynamic and metabolically demanding neural tissues required for vision. Adequate oxygen and nutrient delivery are essential for maintaining tissue function and cellular survival. Over the past decades, extensive research within and beyond the field of ophthalmology has sought to elucidate the mechanisms that govern neurovascular regulation in health and disease. Growing evidence indicates that neurovascular dysfunction plays an important role in both the initiation and progression of glaucoma, a leading cause of irreversible blindness worldwide. Alterations in vascular architecture and blood flow may compromise the metabolic support required by retinal ganglion cells, increasing their vulnerability to injury and degeneration. While neurons possess limited regenerative capacity, the vascular system retains a remarkable degree of plasticity and is therefore amenable to repair. This vascular plasticity presents an opportunity to develop therapeutic strategies aimed at restoring vascular architecture and improving blood flow, complementing existing approaches focused on intraocular pressure reduction, neuroprotection, axonal regeneration, and/or neuronal transplantation. In this review, we summarize the current understanding of neurovascular function in the healthy eye, discuss mechanisms that contribute to vascular compromise in glaucoma, and highlight emerging avenues for promoting vascular regeneration and blood flow recovery. By identifying key knowledge gaps and future research priorities, we aim to outline promising directions for targeting the ocular neurovasculature to preserve retinal ganglion cell function and slow or stop progressive vision loss.

## Introduction

### Healthy ocular neurovasculature: architecture and blood flow regulation

Neural tissues of the visual pathway are supported by a dense and highly specialized vascular network. Under physiological conditions, this vasculature, like that of other regions of the central nervous system, effectively supplies oxygen and nutrients to the metabolically demanding neural tissues required for vision. Ocular neurovasculature maintains relatively stable blood flow despite modest fluctuations in ocular perfusion pressure, a process known as vascular autoregulation (Riva et al., 1981; Prada et al., 2016). In addition to this baseline regulation, blood flow dynamically adjusts to neuronal activity. Visual stimulation, often induced experimentally with a controlled flicker of light, triggers dilation of blood vessels in the retina and optic nerve head (ONH), producing a transient increase in local blood flow (Garhöfer et al., 2002; 2004; Warner et al., 2020). The increased neuronal activity induced by visual stimulation drives the metabolic demand underlying this response, a phenomenon consistently observed in both animal models and human studies (Riva et al., 2005; Mishra et al., 2011). Tightly coupled neuronal activity and local blood flow, first observed in the brain (Roy and Sherrington, 1890; Iadecola, 2017), is known as neurovascular coupling. Effective neurovascular coupling relies on coordinated signaling among multiple cell types, including vascular endothelial cells, pericytes, macroglia, microglia, and neurons, which must effectively work in concert as an interconnected neurovascular unit (Wareham and Calkins, 2020; Alarcon-Martinez et al., 2023). Disruption of signaling within any component of this unit can alter neurovascular dynamics, contributing to a mismatch between vascular supply and neuronal metabolic demand.

### Where do things go wrong in glaucoma?

Vascular compromise is increasingly recognized as a contributing factor in several neurodegenerative diseases, including Alzheimer’s disease (de la Torre and Stefano, 2000; Shi et al., 2021; Li et al., 2024), amyotrophic lateral sclerosis (Garbuzova-Davis et al., 2011; Soldatov et al., 2021; Vautier et al., 2023), and Huntington’s disease (Lin et al., 2013; Amini et al., 2022; Garcia et al., 2022). Vascular dysfunction is also implicated in glaucoma (Wareham and Calkins, 2020; Alarcon-Martinez et al., 2023), a leading cause of irreversible blindness worldwide. In glaucoma, retinal ganglion cells (RGCs), neurons with axons bridging the retina with the rest of the brain via the optic nerve, suffer progressive degeneration. The resulting loss of RGCs leads to irreversible visual impairment. Although the precise mechanisms underlying RGC degeneration remain poorly understood, elevated intraocular pressure (IOP) is strongly associated with glaucomatous damage, with early injury thought to occur at the level of the ONH(Quigley et al., 1981; Sigal and Ethier, 2009; Pitha et al., 2024; Stowell et al., 2017). Historically, glaucoma pathogenesis has been viewed through two core perspectives, with damage resulting from 1) mechanical insult or 2) vascular insufficiency. These perspectives are now largely understood not as opposing explanations, but rather as interrelated processes which each may drive RGC vulnerability and degeneration. Currently, IOP is the only clinically modifiable risk factor for glaucoma and IOP reduction remains the primary therapeutic strategy (Asrani et al., 2024). However, surgical and pharmacological approaches to reduce IOP frequently fail to fully halt vision loss, with approximately half of treated patients continuing to experience meaningful visual decline (Leske et al., 2003; Shin et al., 2024). Additionally, some patients develop progressive optic neuropathy despite having IOP values within the normal range, a condition referred to as normal-tension glaucoma (Killer and Pircher, 2018). These observations indicate that additional mechanisms contribute to disease progression and highlight the need to better understand IOP-independent pathways of RGC vulnerability. Increasing evidence points to vascular compromise as a key factor in the pathophysiology of glaucoma, suggesting that impaired blood flow and vascular regulation contribute critically to early neuronal stress and degeneration.

### Vascular compromise: cause or consequence of neurodegeneration?

Disentangling whether vascular dysfunction is a cause or a consequence of neurodegeneration remains a challenge in ocular neuropathologies. Under physiological conditions, retinal neurons are highly metabolically active and rely on a continuous supply of oxygen and nutrients to sustain their function. In disease, decreased neuronal numbers and/or metabolic activity are expected to reduce tissue metabolic demand, potentially leading to secondary decreases in blood flow and regression of the local vasculature. However, accumulating evidence from both experimental and clinical studies, detailed in the following sections, support the notion that in glaucoma, vascular alterations arise early in the disease process, prior to detectable neurodegeneration. These observations support the possibility that vascular compromise plays a causal role in glaucomatous neuropathy rather than representing a purely secondary consequence of neurodegeneration. Regardless of whether vascular dysfunction acts as an initiating event or emerges as a downstream effector of neuronal injury, evidence supports the existence of vascular compromise as a consistent feature of glaucomatous pathology. Without addressing these vascular deficits, be they primary or secondary, neurodegeneration remains at risk of progression and opportunity for neurorecovery is limited. Given our existing knowledge about structural and functional alterations to ocular vasculature in pathology, vascular regeneration and blood flow recovery arise as separate and synergistic opportunities for therapeutic focus.

## Pathologic changes in vascular structure and function

A wide variety of evidence collected through different methods converge on a consistent conclusion: glaucoma is associated with dysregulated, and often decreased, blood flow as well as reduced vascular density. No single technique has emerged as the standard to measure vascular structure or perfusion in the eye. A non-exhaustive overview of key findings is provided below, noting strengths and limitations of techniques as they relate to vascular alterations in glaucoma.

Basic research studies largely indicate decreased vascular density and impaired blood flow in animal models of experimental glaucoma, often detectable prior to neurodegeneration. In microbead occlusion models of ocular hypertension in mice, the number of acellular capillaries increases markedly at 2 weeks of microbead injection, preceding RGC death (Pitale et al., 2022). These structural alterations are accompanied by an early reduction in basal retinal blood flow and impaired light-evoked neurovascular coupling responses (Pitale et al., 2022). Pericytes in the retina and potentially the ONH as well have emerged as regulators of capillary blood flow (Waxman et al., 2025b), with ocular hypertension-induced rupture of interpericyte tunneling nanotubes and blood flow impairment, preceding RGC death (Alarcon-Martinez et al., 2022). Magnetic resonance imaging further demonstrates a progressive decline in retinal blood flow with age in the DBA/2J genetic mouse model of glaucoma (Lavery et al., 2012). Notably, vascular alterations are also observed in pressure-independent models. For example, in a mouse model of normal tension glaucoma, reduction in peripapillary vascular density was driven by microglia-mediated damage to vascular endothelial cells (Zhang et al., 2025). In a non-human primate model of experimental glaucoma, functional microvascular volume loss in the retinal nerve fiber layer was observed prior to RGC degeneration, as evaluated through neuroretinal rim tissue loss (Dunn et al., 2026).

Clinical studies using non-invasive optical coherence tomography (OCT) and OCT-angiography (OCT-A) imaging support a clear relationship between glaucoma and decreased vascular density in the retina and ONH. OCT-A detects blood flow indirectly through changes in OCT signal between repeated scans acquired at the same retinal location, which reflect erythrocyte motion within perfused vessels^42^. As such, regions lacking OCT-A signal should be interpreted not as definitively devoid of vasculature, but as areas with reduced or absent detectable perfusion at the time of imaging. Within this framework, OCT-A measurements provide an important index of functional microvascular perfusion.

Consistent with this interpretation, a United Kingdom biobank study analyzing retinal fundus photographs from approximately 40,000 participants without a history of glaucoma revealed that reduced retinal vascular density was associated with an increased risk of developing the disease (Chen et al., 2025). Similarly, analysis of approximately 100 patients from the Diagnostic Innovations in Glaucoma Study showed that retinal areas with reduced active vessel density in glaucomatous eyes corresponded closely with locations of visual field deficits as well as thinning of the retinal nerve fiber layer and ganglion cell complex (Yarmohammadi et al., 2016), both established metrics of neurodegeneration. Functional macular vessel density was found to decline significantly faster in glaucoma than ganglion cell complex thickness, a measure of RGC integrity, with rates of change approximately threefold greater. Importantly, reductions in macular vessel density were associated with increasing disease severity (Hou et al., 2020). Additional work suggests that vascular dropout in the ONH may precede similar changes in the macula (Wu et al., 2025). Notably, vascular alterations are also observed in cases of glaucoma without ocular hypertension. Active peripapillary vascular density is significantly reduced in early-stage normal-tension glaucoma compared with healthy controls (Zhang et al., 2025).

Laser speckle flowgraphy (LSFG) (Sugiyama, 2014) enables relative evaluation of blood flow velocity in ocular tissues. Studies utilizing this approach consistently report significantly reduced blood flow velocity at the ONH, both in hypertensive and normotensive glaucoma (Shiga et al., 2016; Mursch-Edlmayr et al., 2018; Kuroda et al., 2020; Gu et al., 2021). The magnitude of ONH blood flow reduction is often closely associated with the degree of structural damage, and decreases in ONH perfusion have been detected prior to the onset of measurable visual field loss (Shiga et al., 2016). Longitudinal analyses further suggest that vascular changes during glaucoma progression are not strictly monotonic. In some cases, reductions in ONH microvascular flow are preceded by a transient increase in blood flow during early stages of disease. This pattern points to complex, stage-dependent vascular responses and the possibility of an early compensatory phase followed by vascular failure, a phenomenon also reported in non-human primate models of experimental glaucoma^48^.

Complementary structural insights have emerged from histologic and high-resolution imaging approaches that visualize the architecture of the ocular microvasculature. These methods provide spatial resolution beyond what is currently achievable with *in vivo* techniques and are particularly valuable for studying the ONH and posterior optic nerve, where imaging can be limited by shadow artifacts, restricted penetration, and reliance on flow-based metrics rather than direct visualization of vascular structure. Representative (Anderson and Braverman, 1976; Lieberman et al., 1976; Hayreh, 2001; Mackenzie and Cioffi, 2008) and serial section analyses (Kang et al., 2018; Lee et al., 2022; Waxman et al., 2022) have helped improve our fundamental understanding of vascular structure and shown that microvascular density within the lamina cribrosa region correlates with RGC axonal volume (Kang et al., 2018). Numerical simulations informed in part by histologic reconstructions predict that elevated IOP has complex influences on lamina cribrosa blood flow and oxygenation (Lu et al., 2024). Beyond histologic sections, emerging approaches for whole ONH and whole eye vascular labeling, optical clearing, and intact 3D imaging at cellular resolution offer promise to improve our understanding of the complex, interconnected network structure of the eye in health and glaucoma (Waxman et al., 2018; 2025a; Darche et al., 2022; 2023; Krasniqi et al., 2024). These approaches offer new opportunities to examine poorly understood vascular alterations that are challenging or impossible to observe *in vivo*, complementing insights that can be gained from longitudinal *in vivo* imaging.

The location of earliest vascular changes in glaucoma has strong relevance for understanding disease onset and progression and identifying approaches for intervention. A substantial body of research points to the lamina region of the ONH as an early site of injury in glaucoma, with downstream consequences for the retina, optic nerve, and visual centers in the brain. However, methods used to assess vascular structure and blood flow are often strongly influenced by the anatomical location and optical properties of the tissues being examined. As a result, absence of detectable microvascular alterations in the lamina region prior to changes observed in the retina or anterior ONH does not necessarily indicate that dysfunction originates in these more superficial tissues. Signals arising from superficial vascular beds are often easier to resolve and quantify and therefore may be overrepresented in current imaging datasets. Advances in approaches to understand this complex vascular structure and function, particularly in deep regions of the ONH, will help clarify the spatial and temporal sequence of vascular dysfunction in glaucoma. Spatially as well as temporally-resolved insights can help guide the development of targeted therapeutic strategies.

## Regenerating vascular architecture

**FIGURE 1:**
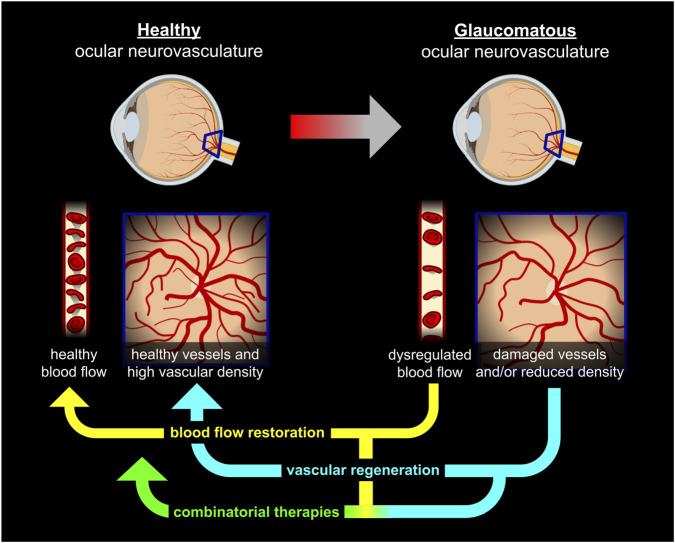
Potential to address glaucomatous vascular compromise through vascular regeneration, blood flow restoration, and combinatorial approaches. Healthy ocular neurovasculature (left) supplies neural tissues of the eye with oxygen and nutrients needed visual function. In both the initial and later stages of glaucoma, there is frequently disruption in blood flow and vascular architecture (right) threatening neuronal insult and degeneration. Therapeutic strategies targeted to restore healthy blood fow and healthy vascular architecture (bottom,) individually and/or in combination, can offer promising paths forward for glaucoma treatment. Made in part with BioRender.

The vascular system is plastic and amenable to repair (Lapi and Colantuoni, 2015; Williamson et al., 2020), unlike neurons, which have limited capacity for regeneration (Fawcett, 2020). This plasticity can offer an approachable pathway to address ocular neuropathologies, compatible with efforts toward neuroprotection, RGC axonal regrowth, and neuronal transplantation. Whether vascular compromise in glaucoma is a cause or a consequence of RGC degeneration, the survival and function of remaining or newly repopulated RGCs require adequate vascular support. Vascular degeneration is positioned to be a key part of a self-reinforcing cycle in which vascular damage promotes or exacerbates RGC loss, reduced RGC density lowers local metabolic demand, and less demand further destabilizes the microvasculature. Strategies that promote vascular regeneration or stabilization provide the opportunity to restore a supportive microenvironment for neuronal survival and repair (Figure 1).

Given the potential importance of vascular stabilization and regeneration in interrupting degenerative feedback between vascular and neuronal loss, considerable attention has focused on molecular pathways that regulate angiogenesis and vascular repair. Among these, vascular endothelial growth factor A (VEGF-A) is a master regulator of angiogenesis, both during development and in post-injury repair (Ma et al., 2012; Lee et al., 2025). In the most prevalent form of glaucoma, primary open angle glaucoma, VEGF-A supplementation has been shown to provide neuroprotection (Foxton et al., 2013). Although VEGF-A plays critical roles in angiogenesis, it is important to note that it plays a host of other roles in signaling beyond its vascular effects. *In vitro* neuronal cultures lacking vasculature demonstrate that VEGF-A protects neurons against hypoxia and glucose deprivation (Jin et al., 2000; Foxton et al., 2013; Wang et al., 2021). Consistent with this, a transgenic mouse line expressing human VEGF-A in neurons exhibited significant RGC protection after optic nerve axotomy, without overt alterations in retinal vascular structure (Kilic et al., 2006). In glaucomatous human donor eyes, ONH astrocytes show reduced VEGF-A expression^64^, suggesting that diminished VEGF-A signaling may contribute to both neuronal compromise and impaired vascular maintenance in this vulnerable region.

At the same time, approaches to prompt vascular regeneration in glaucoma must be carefully balanced with an understanding that pro-angiogenic factors delivered to the eye can have consequences beyond the retina and optic nerve. Neovascular glaucoma is associated with increased VEGF in the eye’s anterior chamber, causing proliferation of fibrovascular tissue within the aqueous humor outflow tract, leading to ocular hypertension, and ultimately optic neuropathy (Tolentino et al., 1996; Tripathi et al., 1998). Untangling the pro-angiogenic benefits and potential risks of VEGF-A in ocular neuropathologies will require strategies that decouple its direct and transient neuroprotective effects from pathological vascular overgrowth while preserving beneficial vascular regeneration.

Equally critical to angiogenesis during vascular regeneration is vessel maturation. An increased density of fragile, leaky, or highly tortuous neovessels with low or dysregulated blood flow offers little promise for functional restoration or neuroprotection. For example, a high density of aberrant retinal vasculature is a hallmark of proliferative diabetic retinopathy, often tempered with local anti-VEGF therapy (Moshfeghi et al., 2024). AAV-mediated VEGF overexpression in the mouse striatum resulted in the formation of enlarged neovessels accompanied by increased vascular permeability and hemorrhage (Stiver et al., 2004). However, after VEGF expression declined, large neovessels underwent remodeling into numerous smaller vessels with prominent pericyte coverage that remained stable and patent for over a year after angiogenesis was induced (Stiver et al., 2004). While VEGF-A is a primary driver of endothelial cell sprouting and proliferation (Gerhardt, 2008; Beheshtizadeh et al., 2023), additional signaling pathways regulate vessel maturation and stabilization. Among these, platelet derived growth factors (PDGFs) play key roles in vessel maturation, stabilization, and pericyte recruitment (Hellström et al., 1999; Hellberg et al., 2010; Li et al., 2022; Zhang Z. et al., 2022; Liu et al., 2025). PDGF-BB in particular, expressed primarily by vascular endothelial cells, is a strong chemoattractant to pericytes through activation of its receptor, PDGFR*β*, leading to pericyte recruitment to immature vessels (Andrae et al., 2008). In addition, pericyte- and astrocyte-derived angiopoietin-1 promotes endothelial cell maturation as well as vascular integrity through tempering the permeability-promoting effects of VEGF (Uemura et al., 2002; Gavard et al., 2008; Brudno et al., 2013; Feng et al., 2025). Together, these findings suggest that the timing, dosage, and spatial control of pro-angiogenic and maturation signals must be carefully coordinated, as successful vascular regeneration will likely require a more nuanced strategy than a simple “more is better” approach to address vascular compromise.

While vascular regression in pathology may reflect a decreased capacity to sustain functional blood flow, regenerating lost vasculature alone is not a fully sufficient goal for neuroprotective outcomes. Restoring effective blood flow within both existing and newly formed vessels therefore represents a critical and complementary therapeutic goal.

## Restoring functional blood flow

As insufficient blood flow to neural tissues can be a direct cause of dysfunction and cell death, ensuring adequate blood supply to the retina and ONH is critical for neuroprotection. All current clinical approaches for glaucoma management are targeted at decreasing IOP, primarily by increasing fluid flow facility through the eye’s aqueous humor outflow tract, a structure with vascular-like properties (Kizhatil et al., 2014; Johnstone et al., 2023; Bilal et al., 2025). Ocular hypertension is known to decrease blood flow in the retina and ONH (Wang et al., 2012; Iwase et al., 2018; Kiyota et al., 2018) while many IOP-lowering treatments are associated with increased blood flow (Gherghel et al., 2008; Iida et al., 2017). It is not always clear whether treatment-induced increases in blood flow are an indirect consequence of IOP reduction or whether pharmacotherapies, often applied as eyedrops, reach the back of the eye at meaningful concentrations and exert effects beyond the outflow structures. A range of factors associated with glaucoma pathology and therapeutics actively modulate vascular diameter through contractile mural cells, which densely populate retinal and ONH vasculature (Rouget, 1873; Schallek et al., 2013; Trost et al., 2019; Waxman et al., 2025b). Agents that induce local vasorelaxation and increase blood flow often exert neuroprotective effects in glaucoma.

What therapeutic strategies have been explored to directly restore blood flow for ocular neuroprotection? Endothelin 1 is a potent vasoconstrictor, commonly present in greater amounts in the posterior chamber of eyes with diabetic retinopathy (Adamiec-Mroczek et al., 2010) and glaucoma (He et al., 2007). PER-001 is an investigational intravitreal implant designed to provide sustained-release of an endothelin receptor antagonist. Phase 2 clinical trials suggest that this approach increases retinal and ONH blood flow with protection of visual function. In glaucoma, it may also offer complementary benefit when combined with IOP lowering therapies (Gray, 2025; Mansberger et al., 2025). Rho-kinase inhibitors, such as ripasudil and netarsudil, are now well-known to decrease IOP in glaucoma. In addition to their effects on aqueous humor outflow, these agents are suggested to exert IOP-independent neuroprotective effects, which may partially occur through vascular smooth muscle relaxation and improved ocular blood flow (Rao and Epstein, 2007; Wang et al., 2023; Reboussin et al., 2024).

As in inherited retinal diseases with known mutation-based causes, AAV-based gene therapy has shown improved visual function and slowed degeneration, with promise from ongoing clinical trials for future vision saving treatments (Butt et al., 2025; Lonfat et al., 2025). In a mouse model of neovascular age-related macular degeneration, a single injection of AAV conferred lasting expression of a VEGF-neutralizing protein, with intraocular concentration of anti-VEGF adjustable through oral supplementation of an activating ligand (Reid et al., 2018). This strategy enables long-term therapeutic modulation without the need for repeated intraocular anti-VEGF injections (Reid et al., 2018). Clinical trials are ongoing for AAV-based anti-VEGF therapy in patients with neovascular age-related macular degeneration (ClinicalTrials.gov, 2026). Similar gene therapy strategies could potentially be adapted for glaucoma by targeting other vascular modulators that regulate perfusion, vascular tone, or endothelial function. Identification of the precise factors and cell types leading to compromised blood flow in ocular neuropathologies can help open doors to confer long-lasting, tunable, and cell-type specific modulation through gene therapy-based approaches.

Organotypic culture systems for human donor eyes provide the opportunity to investigate cellular, tissue, and organ-level dynamics with high experimental flexibility and strong translational relevance. In these systems, oxygenation and nutritional support have most often been applied through bath application of culture media (Abbas et al., 2022; Zhang J. W. et al., 2022; Chan et al., 2024). These approaches provide valuable opportunities to study fundamental ocular physiology as well as testing candidate therapeutics directly in human donor tissues, without the need for time-sensitive and technically challenging cannulation of arterial supply to artificially reinstate blood flow after donation. Recently, a new *ex vivo* approach to maintain postmortem eyes that restores intravascular perfusion shows exciting promise for direct investigation of ocular blood flow physiology and responses to therapeutic candidates (Lohss et al., 2025).

## Conclusions and future directions

Medicine and research have long maintained a primarily neuron-centric view of the central nervous system. Yet, without a robustly functional system of vascular support, neurons are at risk of dysfunction, damage, and death. In the diseased eye, approaches aimed at neuronal protection, regeneration, and transplantation may fall short of achieving sustained vision preservation and restoration if vasculature remains compromised. Improved strategies to regenerate damaged or lost vasculature and to restore blood flow function in glaucoma have promise to address this critical gap. *In vivo* approaches are currently the only means to investigate blood flow, neurovascular unit dynamics, and visual function in the context of an intact eye-brain connection. Improved methods and experimental models are needed to help better understand vascular structure and blood flow regulation in both health and disease. This is particularly true in regions of the visual pathway that are poorly accessible for *in vivo* imaging at cellular or capillary resolution. Ultimately, vascular regeneration and blood flow restoration represent distinct but complementary therapeutic strategies. Their combination may provide a strong opportunity to achieve durable neuroprotection and meaningful preservation of vision in glaucoma and other optic neuropathies.
